# Cerebral bioenergetic differences measured by phosphorus‐31 magnetic resonance spectroscopy between bipolar disorder and healthy subjects living in two different regions suggesting possible effects of altitude

**DOI:** 10.1111/pcn.12893

**Published:** 2019-07-03

**Authors:** Jaeuk Hwang, Lynn E. DeLisi, Dost Öngür, Colin Riley, Chun Zuo, Xianfeng Shi, Young‐Hoon Sung, Douglas Kondo, Tae‐Suk Kim, Rosemond Villafuerte, Diane Smedberg, Deborah Yurgelun‐Todd, Perry F. Renshaw

**Affiliations:** ^1^ Department of Psychiatry University of Utah Salt Lake City USA; ^2^ Department of Psychiatry Soonchunhyang University Hospital Seoul South Korea; ^3^ US Department of Veterans Affairs Boston Healthcare System Brockton USA; ^4^ Brain Imaging Center, McLean Hospital, Department of Psychiatry Harvard Medical School Belmont USA; ^5^ Rocky Mountain Mental Illness Research, Education and Clinical Center US Department of Veterans Affairs Healthcare System Salt Lake City USA; ^6^ Department of Psychiatry Seoul St. Mary's Hospital, The Catholic University of Korea College of Medicine Seoul South Korea

**Keywords:** altitude, bipolar disorder, high‐energy phosphate, magnetic resonance spectroscopy, phosphocreatine

## Abstract

**Aim:**

Increased oxidative stress in cerebral mitochondria may follow exposure to the systemic hypobaric hypoxia associated with residing at higher altitudes. Because mitochondrial dysfunction is implicated in bipolar disorder (BD) pathophysiology, this may impact the cerebral bioenergetics in BD. In this study, we evaluated the cerebral bioenergetics of BD and healthy control (HC) subjects at two sites, located at sea level and at moderate altitude.

**Methods:**

Forty‐three veterans with BD and 33 HC veterans were recruited in Boston (*n* = 22) and Salt Lake City (SLC; *n* = 54). Levels of phosphocreatine, β nucleoside triphosphate (βNTP), inorganic phosphate, and pH over total phosphate (TP) were measured using phosphorus‐31 magnetic resonance spectroscopy in the following brain regions: anterior cingulate cortex and posterior occipital cortex, as well as bilateral prefrontal and occipitoparietal (OP) white matter (WM).

**Results:**

A significant main effect of site was found in βNTP/TP (Boston > SLC) and phosphocreatine/TP (Boston < SLC) in most cortical and WM regions, and inorganic phosphate/TP (Boston < SLC) in OP regions. A main effect analysis of BD diagnosis demonstrated a lower pH in posterior occipital cortex and right OP WM and a lower βNTP/TP in right prefrontal WM in BD subjects, compared to HC subjects.

**Conclusion:**

The study showed that there were cerebral bioenergetic differences in both BD and HC veteran participants at two different sites, which may be partly explained by altitude difference. Future studies are needed to replicate these results in order to elucidate the dysfunctional mitochondrial changes that occur in response to hypobaric hypoxia.

Rates of suicide are increased in higher‐altitude regions of the USA as well as other countries.^1^, ^2^, ^3^, ^4^ Increasing altitude has also been associated with altered local rates of illicit drug use, attention‐deficit hyperactivity disorder, and depressive episodes.^5^, ^6^, ^7^ Although this observation was not replicated in some countries,^8^, ^9^ it is intriguing that altitude may be a crucial factor affecting complex human behaviors, such as suicidal behavior,^10^ in line with reported alterations of various neurotransmitters due to hypobaric hypoxia.^11^ For example, rats exposed to a simulated altitude of 10 000 feet were more likely to display depression‐like behavior associated with neurochemical changes in the frontal lobe.^12^, ^13^

A growing body of literature has suggested that oxidative stress due to mitochondrial dysfunction may be a central pathophysiology of bipolar disorder (BD).^14^ Magnetic resonance spectroscopic studies of the brain have noted abnormal lactate and phosphocreatine (PCr) in BD as well as alterations of intracellular pH and choline‐containing metabolites.^15^ In post‐mortem studies, mitochondria from BD patients are noted to be smaller and atypically distributed in the perinuclear region within cells.^16^ Decreased expression of genes involved in electron transport chain function within mitochondria has also been reported in BD.^17^ In this regard, hypobaric hypoxia in elevated regions may worsen the oxidative stress of BD. This view is in line with the results regarding reduced responses to oxidative stress in the brains of animals exposed to hypobaric hypoxia.^18^ More specifically, the course and natural history of BD may worsen with exposure to the hypobaric hypoxia that accompanies altitude. In fact, altitude of residence has been reported to be a significant predictor of completed suicide in BD.^19^

As altitude increases, the partial pressure of oxygen in the inspired air (PiO_2_) becomes lower. For example, at 4700 feet, the altitude of Salt Lake City (SLC), the atmospheric pressure is around 646 mmHg (compared to 760 mmHg at sea level).^20^ This reduces the pressure gradient that drives oxygen into the bloodstream in the lung alveoli and provides an environment similar to an atmosphere with 18% oxygen concentration.^21^ This hypobaric hypoxia causes a compensatory hyperventilation, hypocapnia, and mild alkalosis; moreover the 15% decrease in atmospheric pressure between SLC and sea level is known to translate to a 20% decrease in the partial pressure of oxygen in arterial blood in healthy human volunteers.^22^ We have previously reported that healthy subjects living in SLC demonstrated a higher pH in the brain compared to subjects in the Boston area near sea level.^23^

In this study, we hypothesized that the cerebral bioenergetic profiles including PCr and adenosine triphosphate (ATP), as well as intracellular pH, would be altered in both BD and healthy control (HC) subjects in environments with markedly different altitudes, which may identify a vulnerability of individuals with BD in elevated altitude. Phosphorus‐31 magnetic resonance spectroscopy (^31^P‐MRS) has been used to assess the phosphate‐bearing metabolites in the regions with both gray and white matter. We compared the cortical intracellular pH, PCr, inorganic phosphate (Pi), β‐nucleic triphosphate (βNTP; primarily ATP in brain), and pH in the subjects recruited at the cities with moderate elevated altitude and near sea level (SLC, Utah and Boston, Massachusetts area).

## Methods

### Subjects

Seventy‐six subjects completed participation across our two study sites (SLC, *n* = 54; Boston, *n* = 22). See Table 1 for demographic details. All participants in the study were US military veterans, aged 18–65 years, who had been a resident of their respective geographic area for a minimum of 2 months, had no history of air travel within the previous 2 months, and had no unstable medical and/or neurological conditions. Subjects were excluded from the study if they: had a current substance or alcohol use disorder, as confirmed by the Structured Clinical Interview for the DSM‐IV‐TR;^24^ were pregnant or currently breastfeeding; had a contraindication to MRI or clinically significant claustrophobia; or had known or suspected mental retardation. Furthermore, subjects had to either have a diagnosis of BD type I as confirmed by the Structured Clinical Interview for the DSM‐IV‐TR or have a lack of any current or past psychiatric diagnoses. Participants also completed the Montgomery–Åsberg Depression Scale (MADRS),^25^ the Young Mania Rating Scale (YMRS),^26^ and the Columbia Suicide Severity Rating Scale (CSSRS).^27^ Based on scores from the MADRS, subjects who had a confirmed BD type I diagnosis were further grouped by mood state: depressed (MADRS score ≥ 18) or euthymic (MADRS score < 18). Participants who were assessed to be in a manic mood state, as indicated by a YMRS score of 12 or greater were not included in the study due to the likelihood of poor compliance during the scan.^28^ The site Institutional Review Boards (University of Utah, McLean Hospital, and Veterans Affairs Healthcare Systems of Salt Lake City and Boston) approved the study's protocol and written informed consent was obtained from all study participants.

**Table 1:** Demographic and clinical information of study participants

Site	Boston (*n* = 22)		Salt Lake City (*n* = 54)		
Diagnosis	HC (*n* = 7)	BD (*n* = 15)	HC (*n* = 26)	BD (*n* = 28)	*P*‐value
Mood state	—	Euthymic: 11 Depressive: 4	—	Euthymic: 17 Depressive: 11	
Age, years (mean ± SD)	51.9 ± 13.7	50.9 ± 9.56	40.0 ± 13.2	46.7 ± 10.8	*P* = 0.014*
Sex, number of males (%)	6 (85.7%)	14 (93.3%)	22 (84.6%)	24 (85.7%)	*P* = 0.92**
MADRS (mean ± SD)	2.00 ± 3.83	7.73 ± 10.0	1.84 ± 3.62	13.0 ± 12.5	*P* = 0.0002*
YMRS (mean ± SD)	1.86 ± 2.91	3.87 ± 5.17	0.73 ± 1.51	4.29 ± 3.97	*P* = 0.003*
Number of items in lifetime CSSRS suicide or behavior	1.29 ± 1.98	2.87 ± 3.02	0.77 ± 1.63	3.93 ± 2.37	*P* < 0.001*
Suicidal ideation intensity rating from lifetime CSSRS	4.00 ± 5.20	6.47 ± 8.16	2.04 ± 4.56	13.1 ± 6.99	*P* < 0.001*

* Mean comparison among four groups using one‐way analysis of variance.

** Fisher's exact test.

BD, bipolar disorder; CSSRS, Columbia Suicide Severity Rating Scale; HC, healthy control; MADRS, Montgomery–Åsberg Depression Rating Scale; YMRS, Young Mania Rating Scale.

### MR image acquisition

Neuroimaging data were acquired using Siemens 3 Tesla whole body MRI systems (Siemens Medical Solutions, Erlangen, Germany) at University of Utah and McLean Hospital. A ^31^P/^1^H double‐tuned volume head coil (Clinical MR Solutions, LLC, Brookfield, WI, USA) was used for phosphorus metabolite data acquisition at each site. Proton decoupled ^31^P spectroscopic imaging data were acquired using a 3‐D magnetic resonance spectroscopy imaging pulse sequence (3‐D MRSI) with the following parameters: field of view (FOV), 20 × 20 × 20 cm^3^; volume of interest, 20 × 20 × 10 cm^3^; receiver bandwidth = 2.5 kHz; repetition time (TR)/echo time (TE) = 3000/2.3 ms; flip angle = 90^°^; average number = 16; vector size = 1024; and matrix scan size = 8 × 8 × 8. To facilitate voxel placement, high‐resolution T_1_‐weighted images (M1) were acquired using a 3‐D magnetization‐prepared rapid gradient echo acquisition (MPRAGE) pulse sequence with the following parameters: TR/TE/inversion time (TI) = 2000/3.37/1100 ms; flip angle = 8^°^; FOV = 256 × 192 × 224 mm^3^; 256 × 192 × 224 matrix size; 1 × 1 × 1 mm^3^ spatial resolution; and bandwidth = 300 Hz/pixel. The anterior–posterior line was identified on the midsagittal images acquired by MPRAGE pulse sequence. In addition, due to the high concentration of water in the brain relative to high‐energy phosphates, suboptimal single‐channel proton coil sensitivity within ^31^P/^1^H dual‐tuned coils is common, reducing structural image quality. Especially in the frontal lobe regions, the dual‐tuned coil's signal‐to‐noise ratio and image contrast were poorer than those acquired using the 12‐channel phased‐array proton head coil. The reduced image contrast in the brain region of interest (ROI) hampers partial voxel volume correction because of less accurate tissue segmentation. Furthermore, tissue‐specific data analysis requires the alignment of the 3‐D MRSI grids at the desired ROI across multiple subjects. Low signal‐to‐noise ratio and poor image contrast reduce the normalization accuracy between T1‐weighted image and standard image. To improve the accuracy of normalization and tissue segmentation, additional 3‐D high‐resolution MPRAGE images (M2) were acquired using a proton‐only 12‐channel phased‐array head coil (TR/TE/TI = 2010/3.57/1100 ms; flip angle = 8^°^; FOV = 256 × 192 × 224 mm^3^; 256 × 192 × 224 matrix size; 1 × 1 × 1 mm^3^ spatial resolution; bandwidth = 260 Hz/pixel).

### ROI selection

**Figure 1:**
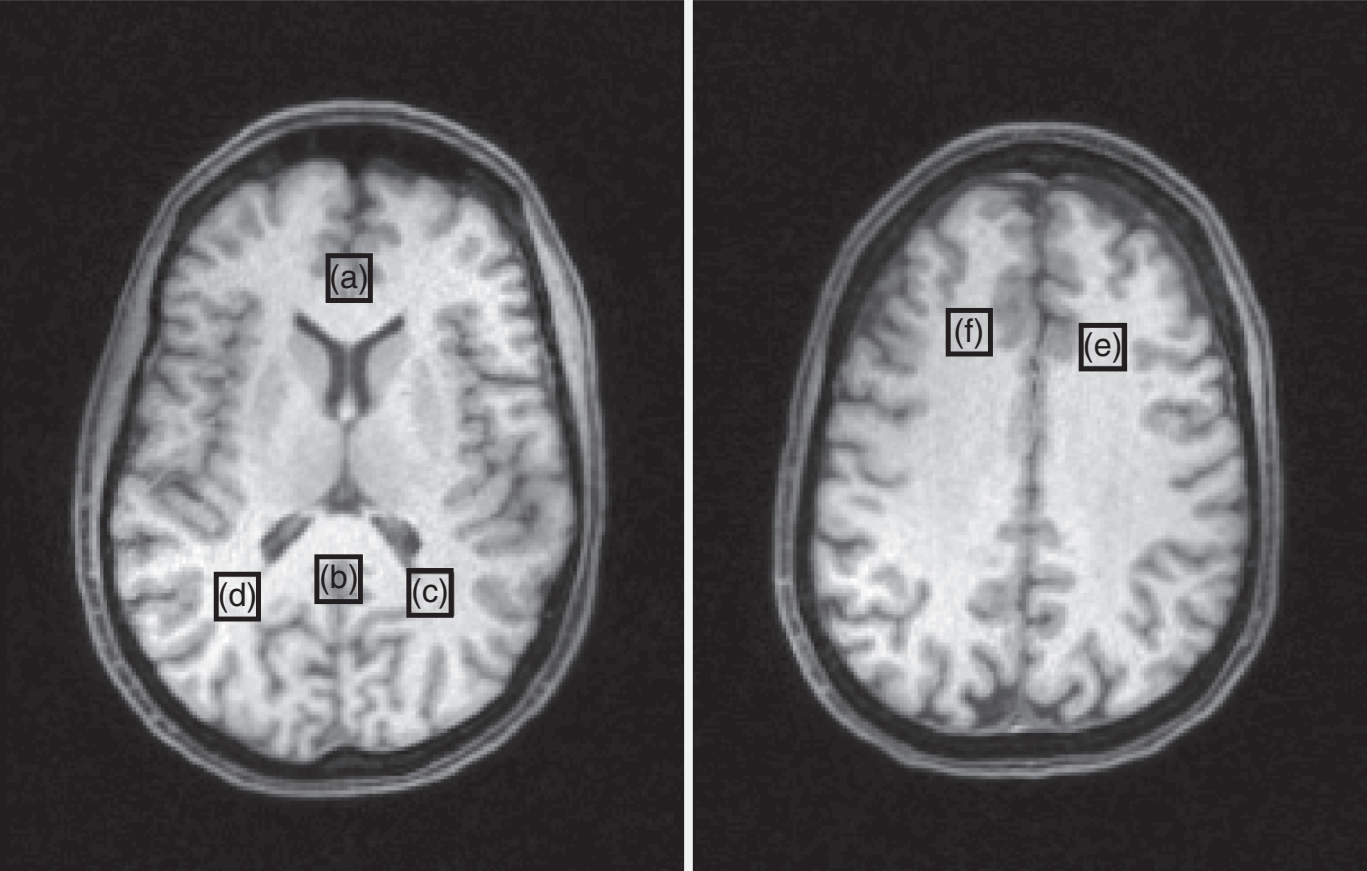
Locations of voxel for phosphorus‐31 magnetic resonance spectroscopy. (a) Anterior cingulate cortex, (b) posterior occipital cortex, (c) left and (d) right occipitoparietal white matter, (e) left and (f) right prefrontal white matter.

In the present study, multiple ROI selections, including anterior cingulate cortex (ACC), posterior cingulate cortex (POC), bilateral prefrontal white matter (WM), and occipitoparietal (OP) WM, are shown in Figure 1. To obtain consistent tissue location across participants, the tissue‐specific voxel center was determined in the Montreal Neurological Institute (MNI) standard image. Second, in order to align M1 with M2 structural images with clear boundary contours, M1 images acquired using ^31^P/^1^H dual‐tuned head coil were registered into M2 images of the same brain. The affine transformation matrix (T_M1‐ > M2_) from M1 to M2 was inverted and then T_M2‐ > M1_ (the inverted T_M1‐ > M2_) was applied onto M2 to obtain registered M1 (M1^*^) in the M1 subject‐native space. Third, the normalization of the M1^*^ image into the MNI template space was performed to achieve a warping field map using the FNIRT tool of FSL (FMRIB Software Library, Release 4.1; Oxford, England). The inverse warping field image is used to map the voxel center determined in the first step from MNI space to subject native space. Finally, according to the property of MRSI grid re‐shifting with an additional linear phase application in the k‐space domain, all MRSI grids were aligned with respect to the ROI center in the subject‐native space.^29^

### MRS spectra processing

All ^31^P spectra were preprocessed using a MATLAB‐based application (MathWorks, Inc., Natick, MA, USA). The 3‐D MRSI matrix was interpolated to 16 × 16 × 16 and a 75% Hamming filter was applied to reduce signal contamination in neighboring voxels. Spectra from the desired ROI location within the aligned MRSI grid were extracted and then apodized with 2‐Hz Lorentzian line broadening before Fast Fournier Transform. The zero‐ and first‐order phase corrections were performed on all spectra. The signal intensity of each metabolite was obtained using the Advanced Method for Accurate, Robust and Efficient Spectral Fitting of MRS data with use of prior knowledge (AMARES) fitting algorithm within the software application jMRUI 6.0.^30^ Metabolite concentrations were calculated as a percentage of the total phosphorus (TP) signal acquired from the respective ROI.

### Brain structural image segmentation

To evaluate the influence of brain tissue components within the ROI, the M1^*^ images were segmented into gray matter (GM), WM, and cerebrospinal fluid (CSF) using FSL. Brain tissue images were extracted by removing the outer skull and scalp surfaces using the Brain Extraction Tool of FSL. Finally, the FAST/FIRST tool was utilized to calculate the segmented tissue percentage in the ROI. Coregistration between the spectroscopic ROI and the segmented image was performed with a user‐developed MATLAB program.^31^, ^32^

### Reliability of the scans between sites

The comparability of spectral data across the two study sites has been validated using phantoms as reported in an earlier publication.^23^ Although the same type of scanner manufactured by Siemens and dual‐tuned RF coil were used at both sites, we also monitored the quality of MR spectroscopic imaging by performing repeated ^31^P‐MRS scans at both sites. Five healthy subjects living in SLC traveled to Boston, and were scanned at both sites within a 24‐h period in order to assess and establish quality control. The results indicated that upper range of within‐subject coefficient of variation (COV) was 14.7%, which belongs to desirable levels of COV.^33^ Explorative analyses of the reliability scans are presented in Table S1.

### Statistical analysis

Statistical analyses were conducted using stata 15 (Stata Corporation, College Station, TX, USA). Fisher's exact and one‐way analysis of variance (ANOVA) tests were employed to compare the composition of sex and the means of continuous variables in demographic and clinical information among the four groups. As there was a significant difference in age among the groups (*P* = 0.014), this difference has been taken into consideration in further analyses. In addition, the composition of CSF in the ACC voxel was significantly different across the groups (Table S2). Since CSF contributes much less to the phosphate signals of ^31^P‐MRS than GM or WM, the ratio of CSF was covaried in the analyses of metabolites from the ACC voxel. Analysis of covariance (ancova) was used to assess the altitude (place), the diagnosis, and their interaction effects on the ^31^P‐MRS metabolites, including covariates. Due to a small sample size for HC subjects in Boston, the 95% confidence interval (CI) of *P*‐value was estimated using a non‐parametric random permutation test with 5000 iterations of ancova tests. After ancova was applied, adjusted prediction of mean value with 95%CI was estimated by observation information matrix models using maximum likelihood. Correlation analyses between two continuous variables were conducted using Pearson's correlation. A *P*‐value < 0.05 was regarded as statistically significant. Then, the Benjamini–Hochberg procedure of false discovery rate was used to reduce false positive rates arising from multiple comparison issues of the mean comparison of the metabolites in all the voxels.^34^

## Results

Detailed demographic information and clinical characteristics are presented in Table 1. There were significant differences in MADRS‐, YMRS‐, and CSSRS‐related measurements across the four groups (one‐way ANOVA, d.f. = 3, 72, *F* > 5.19, *P* < 0.003), mostly driven by the differences between BD and HC. Within BD subjects, there were no significant differences in terms of MADRS, YMRS, and number of items in lifetime CSSRS suicide behavior between the patients from the two sites (*t*‐test, d.f. = 41, *t* < 1.43, *P* > 0.16). BD subjects in SLC had greater suicidal ideation intensity ratings than those in Boston (*t*‐test, d.f. = 41, *t* = 2.82, *P* = 0.0074). History of alcohol use of the subjects is presented in Tables S3 and S4.

In the regions of gray matter, a main effects analysis identified site effects in βNTP/TP (*F* > 11.83, *P* < 0.001) and PCr/TP (*F* > 7.43, *P* < 0.008) in both the ACC and the POC, and Pi/TP (*F* = 12.67, *P* = 0.0007) in the POC. In predicted estimation of mean value adjusted by diagnosis and/or covariates, the subjects living in SLC had 7.66% and 6.02% higher PCr levels within ACC and POC, respectively, whereas 19.0% and 22.7% lower βNTP levels (a proxy measure of adenosine triphosphate in the brain) of ACC and POC were found in the subjects from SLC compared to those from Boston. The predicted Pi of POC was 14.8% higher in the subjects from SLC. A lower pH within POC in BD subjects was also observed relative to HC subjects as a main‐effects analysis of diagnosis (*F* = 7.33, *P* = 0.009). Upper limits of 95%CI for all the *P*‐values of ancova tests were estimated below 0.05. Detailed comparisons of ^31^P‐MRS results in ACC and POC are presented in Table 2.

**Table 2:** Phosphorus‐31 magnetic resonance spectroscopy results in anterior cingulate and posterior occipital cortices

Site	Boston(*n* = 22)		Salt Lake City(*n* = 54)		Main effects*		Interaction*Site × Diagnosis
Diagnosis	HC(*n* = 7)	BD(*n* = 15)	HC(*n* = 26)	BD(*n* = 28)	Site	Diagnosis	
	Mean ± SD95%CI†	Mean ± SD95%CI†	Mean ± SD95%CI†	Mean ± SD95%CI†	*P*‐value		
					Upper 95%CI‡ *P*‐value		
Anterior cingulate cortex							
PCr/TP	0.143 ± 0.018	0.154 ± 0.012	0.161 ± 0.016	0.162 ± 0.016	**0.008** **	0.14	0.20
	0.132–0.155	0.146–0.163	0.154–0.167	0.156–0.167	0.010		
βNTP/TP	0.102 ± 0.031	0.091 ± 0.019	0.083 ± 0.017	0.079 ± 0.018	**0.001** **	0.29	0.36
	0.090–0.119	0.083–0.105	0.072–0.089	0.072–0.087	0.002		
Pi/TP	0.055 ± 0.018	0.049 ± 0.009	0.052 ± 0.012	0.051 ± 0.020	0.47	0.24	0.73
	0.041–0.064	0.038–0.054	0.048–0.061	0.045–0.056			
pH	7.005 ± 0.054	7.017 ± 0.029	7.021 ± 0.031	7.016 ± 0.045	0.64	0.37	0.64
	6.984–7.041	7.006–7.047	6.996–7.028	7.003–7.031			
Posterior occipital cortex							
PCr/TP	0.176 ± 0.023	0.178 ± 0.010	0.183 ± 0.013	0.186 ± 0.013	**0.005** **	0.73	0.94
	0.164–0.184	0.169–0.182	0.180–0.190	0.181–0.191	0.007		
βNTP/TP	0.098 ± 0.026	0.097 ± 0.014	0.080 ± 0.014	0.074 ± 0.018	**<0.0001** **	0.47	0.75
	0.087–0.112	0.089–0.106	0.072–0.085	0.068–0.080	0.0007		
Pi/TP	0.053 ± 0.009	0.055 ± 0.005	0.059 ± 0.008	0.063 ± 0.009	**0.0007** **	0.23	0.81
	0.046–0.059	0.050–0.059	0.057–0.063	0.060–0.066	0.001		
pH	7.038 ± 0.012	7.029 ± 0.009	7.040 ± 0.013	7.031 ± 0.096	0.96	**0.009** **	0.74
	7.031 – 7.048	7.025–7.036	7.034–7.043	7.027–7.035		0.009	

Bold indicates the significant results (*P* < 0.05).

* ancova including age and composition of cerebrospinal fluid in the voxel (anterior cingulate cortex) or age only (posterior occipital cortex) as continuous covariates.

** Significant after Benjamini–Hochberg procedure of false discovery rate was applied.

† Adjusted prediction of mean value with 95%CI estimated by observation information matrix models using maximum likelihood after ancova applied.

‡ 95%CI of *P*‐value estimated using non‐parametric random permutation test with 5000 iterations of ancova tests.

ancova, analysis of covariance; BD, bipolar disorder; CI, confidence interval; HC, healthy control; βNTP, β nucleoside triphosphate; PCr, phosphocreatine; Pi, inorganic phosphate; TP, total phosphorus signal.

**Figure 2:**
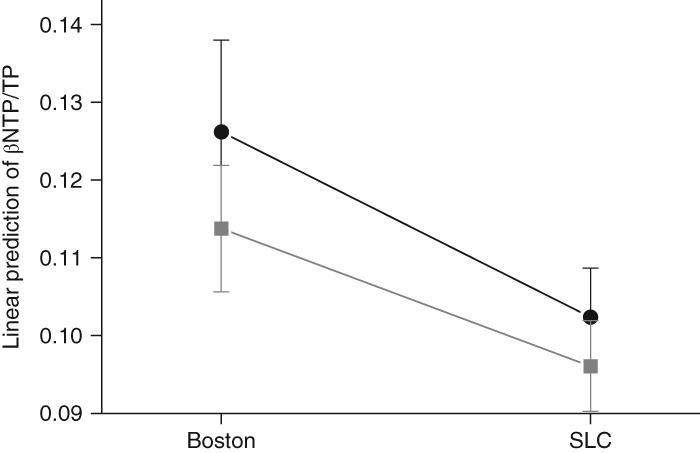
Predictive mean values with 95% confidence intervals of β nucleoside triphosphate (βNTP)/total phosphorus signal (TP) in right prefrontal white matter region adjusted by main factors and age. (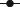) Bipolar disorder. (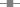) Healthy control. SLC, Salt Lake City.

In the regions of WM, the differences of βNTP/TP and PCr/TP by site were similar to those within the ACC and POC. Aside from right prefrontal WM, lower βNTP/TP (*F* > 13.78, *P* < 0.0004) by 17.5%–23.1% in predicted value and higher PCr/TP (*F* > 4.04, *P* < 0.048) by 4.67%–6.06% were observed in the subjects from SLC relative to those from Boston across the regions. In right prefrontal WM, both altitude and diagnosis effects were found in βNTP/TP (*F* > 5.08, *P* < 0.027; Fig. 2), while not in PCr/TP (*F* < 3.07, *P* > 0.08). Like POC, higher ratios of Pi/TP (*F* > 4.71, *P* < 0.033) by 12.6% and 7.79% (left and right, respectively) were observed in bilateral OP WM of the subjects from SLC relative to those from Boston, whereas lower pH of right OP WM (*F* = 14.0, *P* = 0.0004) only was observed in the subjects with BD relative to those in the HC group. Upper limits of 95%CI for all the *P*‐values of ancova tests were estimated below 0.05 except PCr level of left prefrontal WM. However, the PCr/TP ratio in the left prefrontal, and PCr/TP and Pi/TP ratios for the right OP WM regions between the sites and βNTP/TP in right prefrontal WM between diagnosis did not remain significant after multiple comparison correction. Detailed comparisons of ^31^P‐MRS results in the regions of WM are presented in Table 3.

**Table 3:** Phosphorus‐31 magnetic resonance spectroscopy results in the regions of bilateral prefrontal and occipitoparietal white matter

Site	Boston(*n* = 22)		Salt Lake City(*n* = 54)		Main effects*		Interaction*Site × Diagnosis
Diagnosis	HC(*n* = 7)	BD(*n* = 15)	HC(*n* = 26)	BD(*n* = 28)	Site	Diagnosis	
	Mean ± SD	Mean ± SD	Mean ± SD	Mean ± SD	*P*‐value		
	95%CI†	95%CI†	95%CI†	95%CI†	Upper 95%CI‡ *P*‐value		
Left prefrontal WM							
PCr/TP	0.172 ± 0.021	0.171 ± 0.017	0.176 ± 0.012	0.179 ± 0.014	**0.048**	0.95	0.77
	0.160–0.182	0.162–0.177	0.172–0.183	0.173–0.184	0.06		
βNTP/TP	0.116 ± 0.020	0.116 ± 0.015	0.101 ± 0.024	0.096 ± 0.018	**0.0004** **	0.73	0.89
	0.103–0.134	0.107–0.128	0.091–0.107	0.089–0.104	0.002		
Pi/TP	0.051 ± 0.008	0.050 ± 0.008	0.052 ± 0.009	0.051 ± 0.009	0.43	0.61	0.66
	0.043–0.057	0.045–0.054	0.049–0.056	0.047–0.054			
pH	7.018 ± 0.015	7.030 ± 0.009	7.026 ± 0.019	7.021 ± 0.019	0.66	0.45	0.13
	7.005–7.034	7.021–7.042	7.017–7.033	7.014–7.028			
Right prefrontal WM							
PCr/TP	0.172 ± 0.026	0.168 ± 0.015	0.172 ± 0.013	0.176 ± 0.016	0.084	0.86	0.59
	0.158–0.181	0.158–0.174	0.168–0.181	0.170–0.182			
βNTP/TP	0.127 ± 0.014	0.115 ± 0.012	0.101 ± 0.015	0.096 ± 0.017	**<0.0001** **	**0.027**	0.47
	0.114–0.138	0.106–0.122	0.096–0.109	0.090–0.102	0.0007	0.031	
Pi/TP	0.049 ± 0.007	0.048 ± 0.010	0.051 ± 0.008	0.054 ± 0.010	0.069	0.86	0.52
	0.042–0.055	0.043–0.052	0.048–0.055	0.050–0.057			
pH	7.026 ± 0.034	7.028 ± 0.018	7.027 ± 0.021	7.024 ± 0.024	0.92	0.91	0.67
	7.008–7.043	7.015–7.039	7.018–7.037	7.015–7.033			
Left occipitoparietal WM							
PCr/TP	0.168 ± 0.024	0.171 ± 0.012	0.175 ± 0.012	0.179 ± 0.013	**0.007** **	0.61	0.83
	0.156–0.176	0.162–0.176	0.172–0.183	0.173–0.183	0.009		
βNTP/TP	0.103 ± 0.027	0.108 ± 0.014	0.088 ± 0.014	0.081 ± 0.018	**<0.0001** **	0.86	0.25
	0.091–0.117	0.010–0.118	0.080–0.094	0.074–0.087	0.0007		
Pi/TP	0.054 ± 0.006	0.051 ± 0.004	0.057 ± 0.008	0.059 ± 0.006	**0.0008** **	0.92	0.17
	0.049–0.058	0.047–0.054	0.055–0.060	0.057–0.062	0.003		
pH	7.035 ± 0.016	7.029 ± 0.009	7.037 ± 0.014	7.033 ± 0.012	0.99	0.23	0.48
	7.028–7.046	7.024–7.037	7.030–7.040	7.028–7.038			
Right occipitoparietal WM							
PCr/TP	0.170 ± 0.024	0.171 ± 0.010	0.174 ± 0.011	0.179 ± 0.011	**0.022**	0.50	0.79
	0.159–0.178	0.163–0.176	0.170–0.180	0.174–0.183	0.026		
βNTP/TP	0.110 ± 0.022	0.106 ± 0.012	0.088 ± 0.014	0.082 ± 0.013	**<0.0001** **	0.21	1.00
	0.101–0.123	0.100–0.115	0.081–0.093	0.077–0.087	0.0007		
Pi/TP	0.051 ± 0.008	0.056 ± 0.006	0.057 ± 0.007	0.057 ± 0.008	**0.033**	0.30	0.18
	0.045–0.056	0.051–0.059	0.055–0.061	0.054–0.060	0.036		
pH	7.045 ± 0.007	7.033 ± 0.014	7.042 ± 0.015	7.028 ± 0.010	0.17	**0.0004** **	0.93
	7.036 – 7.056	7.027–7.040	7.036–7.046	7.024–7.033		0.001	

Bold indicates the significant results (*P* < 0.05).

* ancova, including age as a continuous covariate.

** Significant after Benjamini–Hochberg procedure of false discovery rate was applied.

† Adjusted prediction of mean value with 95%CI estimated by observation information matrix models using maximum likelihood after ancova applied.

‡ 95%CI of *P*‐value estimated using non‐parametric random permutation test with 5000 iterations of ancova tests.

ancova, analysis of covariance; BD, bipolar disorder; CI, confidence interval; HC, healthy control; βNTP, β nucleoside triphosphate; PCr, phosphocreatine; Pi, inorganic phosphate; TP, total phosphorus signal; WM, white matter.

No significant interaction was observed between site and diagnosis factors across the two sites. In correlation analyses between clinical ratings, including MADRS, YMRS, and CSSRS, and metabolite levels within the BD group at each site, we did not find any results with statistical significance.

## Discussion

The present results are, to the best of our knowledge, the first report of a study intended to explore ^31^P‐MRS high‐energy cerebral metabolites in BD and HC subjects who reside at different altitudes. While it would be ideal to conduct the study by scanning the same BD and HC subjects at both sites, while allowing for a substantial period of acclimatization, that was considered logistics‐ and cost‐prohibitive. We previously reported that altitude is a risk factor for suicide,^19^ and also reviewed the neuroimaging evidence for mitochondrial dysfunction in BD^15^; therefore, this study was designed to compare and contrast the ^31^P‐MRS high‐energy neurometabolites in BD and HC, at sea level versus moderate altitude. We found site effects on the brain bioenergetic metabolites in this study. Particularly, PCr was increased and βNTP was decreased in most brain regions of subjects living in SLC, which is at moderately elevated altitude (1432 m; 4700 ft) relative to in Boston, near sea level (43 m; 141 ft). We also found that decreased βNTP levels in right prefrontal WM were affected by both site and BD. In addition, there were increased Pi levels within the POC in the subjects from SLC and decreased pH values of occipital lobe in the subjects with BD. In line with the previous literature regarding suicide rates in the USA, BD subjects from SLC showed higher suicidal ideation intensity rating than those from Boston.

As hypothesized, altitude differences between the sites may have led to mild hypobaric hypoxic stress on the brains of the SLC residents. However, if the current results represent the long‐term, chronic adaptation in brain mitochondrial functioning to altitude‐associated hypobaric hypoxia, our hypotheses should fulfill the following assumption. We assumed that mild hypobaric hypoxia in the elevation of about 1.5 km would be enough to provide altitude‐related changes in the human brain. Clearly, mountain sickness, active acclimatization response, and declining cognitive functions are not observed in the subjects living in SLC.^35^, ^36^ However, it has been speculated that modest differences in altitude might play a role in human health.^37^ For example, it has been reported that obesity and diabetes are less prevalent^38^, ^39^, ^40^ and that patients with coronary heart disease would live longer in areas under 1.5‐km elevation.^41^ Animal experiments employing a simulated altitude of about 1370 m (4500 ft) also supported that consistent mild hypobaric hypoxia will produce changes in behavior and antidepressant responses.^13^, ^42^ It is also known that residents in SLC have higher pH values in arterial blood than those at sea level.^22^ Taken together, it seems plausible that environments with mild levels of elevation could affect human body systems.

Although differences in altitude may be responsible for the current findings, other confounding factors may also be important. For example, low consumption of alcohol was observed in Utah relative to in Massachusetts.^43^ Alcohol consumption is known to affect the cerebral bioenergetic metabolites measured by ^31^P‐MRS.^44^ As presented in Tables S2 and S3, self‐reported days of alcohol drinking over the prior months did not differ significantly between sites, although there was a non‐significant trend toward fewer total years of alcohol drinking in the subjects from SLC relative to those of Boston. Although we tried to address the confounding effects of alcohol consumption in the recruitment and acquisition of data, we could not rule them out completely, which could weaken the relationship between altitude and cerebral bioenergetics in our speculations. However, other regional differences in smoking habits, caffeine consumption, food preferences, and so forth, which may affect the results in this study, were not measured objectively in the participants. Potential effects of those confounding factors should be considered when interpreting the results.

Hypobaric hypoxia as a consequence of elevated altitude could be connected to an increased risk for suicide and depression through two molecular pathways.^10^ First, hypoxia could lead to suppressed mitochondrial function,^45^ and result in altered cerebral bioenergetics. Second, decreased cerebral serotonin levels, which have been therapeutic targets of antidepressants as well as markers for suicide, may be observed under hypoxic conditions.^46^, ^47^ In animal studies, it has been shown that levels of serotonin as well as other monoamine neurotransmitters in the brain were decreased as a consequence of exposure to hypoxic conditions.^48^ Additionally, it has been reported that hypoxia could inhibit the activity of tryptophan hydroxylase, which produces serotonin from its precursor, 5‐hydroxy‐tryptophan.^49^, ^50^

In light of altered cerebral bioenergetics in BD, Kato and coworkers reported indicating that decreased levels of PCr in the frontal lobes might be associated with the pathophysiology of BD.^51^ On the other hand, recently it has been reported that unmedicated BD patients had higher levels of PCr than medicated patients.^52^ Similarly, recent work by Harper and coworkers suggested that increased PCr in gray matter of unmedicated patients with major depression may indicate a state marker of depressed mood.^53^ In this regard, the current findings, increased PCr levels of people living in high altitudes, may reflect the bioenergetic vulnerability of the brain to mood disorders.

In the WM of the right frontal lobe, both altitude and diagnosis effects in the levels of βATP were found. This was similar to the findings from a ^31^P‐MRS study with adolescents with BD.^54^ Dudley and coworkers reported decreased ATP in WM of the right hemisphere in adolescents with BD and its probable association with the downregulation of a brain isoform of creatine kinase (CK) in BD.^54^ Of note, several neuroimaging studies have reported that right hemisphere abnormalities are associated with BD,^55^ which suggests a right hemispheric dominance of dysfunctional mood regulation. In addition, we found lower intracellular pH in BD, which was also in line with the previous literature.^15^ Glycolytic shift to compensate mitochondrial dysfunction in BD could produce more lactate, which can lower the intracellular pH. Another possible factor that affects intracellular pH may be mild hyperventilation under hypoxic condition. High‐altitude residents showed higher levels of pH in both arterial blood and brain than did sea‐level residents.^22^, ^23^ This opposite directional drive in pH may dim the pH differences in the study population.

Altered levels of cerebral high‐energy phosphate compounds were supposed to be elucidated by the following molecular mechanisms. First, hypoxia inducible factor (HIF) may play a certain role in a molecular pathway for increasing PCr under hypoxia. HIF is a key molecule helping the cells to survive in a hypoxic environment.^56^ In the previous studies with animals and *in vitro* experiments, it was reported that HIF‐2β had a high affinity for promoter areas of brain and muscle isoforms of CK enzyme as well as creatine (Cr) transporter genes, which suggested indirect evidence that HIF‐1 may serve as a transcriptional factor for genes involved in Cr metabolism.^57^ These findings imply that HIF may enhance the PCr shuttle system as a compensatory response to decreased ATP production in hypoxia, and may be fulfilled through increased expression of CK enzymes and the Cr transporter. Second, modification of Cr kinase by reactive oxygen species under mild hypoxia may lead to the failure of the replenishment of decreased ATP from PCr in cytosol, which would be transferred to adenosine diphosphate in order to maintain physiologic levels of ATP through the activity of the brain isoform of CK (CK‐BB).^58^ A similar profile of bioenergetic molecules with the current findings (increased PCr and decreased ATP levels) has been reported in R6/2 transgenic mice, which are thought to be an animal model of Huntington disease.^59^ In this study, increased levels of PCr in the mice brain were associated with decreased activity of CK‐BB. Additionally, the progression of Alzheimer's disease is accompanied by the loss of CK‐BB activity as well as increased levels of PCr.^60^, ^61^ Decreased activity of CK‐BB in Alzheimer's disease may be caused by the oxidative modification of CK‐BB in the post‐translational stage through ROS, which could be generated in hypoxic conditions.^62^, ^63^

The current study has several limitations. The number of healthy subjects assessed in Boston was relatively small, which weakens the statistical power. Also, the inclusion of both euthymic and depressive BD patients might have affected the results. There have been a few reports regarding the differences in bioenergetic profile measured by ^31^P‐MRS and lateralization depending on mood states.^64^ Due to the small sample sizes in each mood state, we could not reliably conduct the analyses across the mood states. Most BD subjects were treated with psychotropic medications when they participated in the study. Psychotropic medications, including lithium, could affect the levels of PCr in the brain.^65^ Male dominance and US veterans for study participants may be other limitations that undermine the generalizability of the study results. According to recent reports, the number of veterans with BD receiving Veterans Affairs health‐care services increased rapidly to nearly 130 000 in 2014.^66^ The most serious consequence of BD is suicide and BD itself is an important risk factor for suicide among veterans.^67^ In veterans, progressively increasing suicide rates have been regarded as a major public health concern and it has been reported that suicide in veterans was more common than in the US general population.^68^ In addition to suicide, BD is known to devastate the quality of life for veterans, since it has been associated with serious medical comorbidities at earlier ages, unemployment, and homelessness as well as incarceration among veterans.^69^, ^70^, ^71^ While a study population consisting only of veterans could be considered a limitation, investigation of the pathophysiology underpinning the development and exacerbation of BD in veterans seems important and timely. Whereas the ratio of CSF in the voxels of interest was taken into consideration statistically for the analyses, levels of PCr and ATP in this study were supposed to be influenced by the composition of GM and WM.^72^ However, as there is no extant method for partial volume correction in ^31^P‐MRS, like proton MRS, the composition of GM and WM has not been considered in this study.

This is the first study examining the effects of geographic area, and specifically altitude of residence, on the pathophysiology of BD. While our findings are difficult to interpret in isolation, they fit into a model of mitochondrial dysfunction in BD when considered in the context of what is known about hypoxia, suicide, and mood disorders. For example, it has been reported that patients with hypoxic chronic medical conditions are at increased risk for suicide, and that this risk increases with altitude.^73^ Pulmonary functions could be compromised paradoxically by elevation of altitude.^74^, ^75^, ^76^ In addition, patients with BD demonstrate reduced cerebral blood flow to critical brain structures,^77^ which may further reduce oxygen delivery, adding to hypoxic stress in BD at increased altitude. Given the evidence for mitochondrial dysfunction in BD,^14^, ^78^ the multifaceted additional burdens of hypoxia that are known to be associated with altitude warrant further investigation and this work has the potential to have significant translational implications.

The results of the present study are also consistent with the published evidence that increased suicide rates are associated with mountainous areas of the USA, an association that, notably, has been replicated by investigators working in multiple other countries,^1^, ^4^, ^79^, ^80^, ^81^ which may be plausibly linked with altered brain bioenergetics in the cerebral cortex of both BD and healthy subjects, possibly reflecting altitude‐related changes in the brain. These changes in brain chemistry may serve as biomarkers relevant to altitude that may contribute to the vulnerability of individuals who suffer from, or are at risk for the development of, BD. However, a more detailed understanding of how these changes interact with the underlying genetic and neurochemical underpinnings of BD will require further work in larger cohorts of patients and controls.

## Disclosure statement

No authors have any conflicts of interest to report.

## Author contributions

All authors contributed to and approved this version of the manuscript. J.H. conducted the statistical analyses and wrote the manuscript. C.R. and D.S. recruited participants for the study, conducted clinical assessments and study measures, and contributed to the writing of the manuscript. X.S., Y‐H.S., and C.Z. acquired and processed the magnetic resonance spectroscopy imaging data. R.V. facilitated the acquisition of magnetic resonance spectroscopy data at the Brockton site. D.Y‐T., D.K., and D.Ö. served as coinvestigators and clinicians on the project. T‐S.K. contributed to study design. P.F.R. and L.E.D. were principal investigators of the study and provided oversight for all study staff, and direction for the collection, analysis, and interpretation of all data.

## Supporting information


**Table S1.** Exploratory mean comparisons of repeated measurements (five subjects) from phosphorus‐31 magnetic resonance spectroscopy for the reliability of the scans between sites
**Table S2.** Tissue‐composition comparisons in each voxel among the groups
**Table S3.** Self‐reporting total years of alcohol drinking in lifetime
**Table S4.** Self‐reporting days of alcohol drinking in a week during the last 6 months
