# Supplementary material for: Cerebral bioenergetic differences measured by phosphorus‐31 magnetic resonance spectroscopy between bipolar disorder and healthy subjects living in two different regions suggesting possible effects of altitude
Source: Psychiatry Clin Neurosci. 2019 Jul 3;73(9):581–9. doi: 10.1111/pcn.12893 (PMC6771782; doi:10.1111/pcn.12893)
Supplement: Supplementary file 1 — Table S1. Exploratory mean comparisons of repeated measurements (five subjects) from phosphorus‐31 magnetic resonance spectroscopy for the reliability of the scans between sites Table S2. Tissue‐composition comparisons in each voxel among the groups Table S3. Self‐reporting total years of alcohol drinking in lifetime Table S4. Self‐reporting days of alcohol drinking in a week during the last 6 months [file PCN-73-581-s001.docx]

**Supplementary Table 1. Exploratory mean comparisons of repeated measurements (5 subjects) from phosphorus 31 magnetic resonance spectroscopy for the reliability of the scans between sites.**

| Voxel Location |  | Boston | Salt Lake City | N=5 |
| --- | --- | --- | --- | --- |
|  |  | Mean ± SD | Mean ± SD | p value^*^ |
| Anterior cingulate cortex | PCr/TP | 0.150 ± 0.009 | 0.159 ± 0.007 | 0.039 |
|  | βNTP/TP | 0.101 ± 0.004 | 0.112 ± 0.008 | <0.001 |
|  | Pi/TP | 0.055 ± 0.006 | 0.052 ± 0.012 | 0.508 |
|  | pH | 7.012 ± 0.035 | 7.015 ± 0.022 | 0.757 |
| Posterior occipital cortex | PCr/TP | 0.168 ± 0.006 | 0.165 ± 0.012 | 0.520 |
|  | βNTP/TP | 0.101 ± 0.002 | 0.106 ± 0.002 | <0.001 |
|  | Pi/TP | 0.060 ± 0.006 | 0.057 ± 0.005 | 0.147 |
|  | pH | 7.035 ± 0.011 | 7.031 ± 0.008 | 0.304 |
| Left prefrontal white matter | PCr/TP | 0.157 ± 0.011 | 0.163 ± 0.011 | 0.141 |
|  | βNTP/TP | 0.120 ± 0.012 | 0.130 ± 0.013 | 0.002 |
|  | Pi/TP | 0.051 ± 0.007 | 0.049 ± 0.006 | 0.506 |
|  | pH | 7.023 ± 0.026 | 7.020 ± 0.005 | 0.832 |
| Right prefrontal white matter | PCr/TP | 0.156 ± 0.009 | 0.157 ± 0.009 | 0.785 |
|  | βNTP/TP | 0.120 ± 0.007 | 0.126 ± 0.013 | 0.328 |
|  | Pi/TP | 0.055 ± 0.010 | 0.055 ± 0.011 | 0.977 |
|  | pH | 7.020 ± 0.014 | 7.028 ± 0.019 | 0.122 |
| Left occipito-parietal white matter | PCr/TP | 0.163 ± 0.006 | 0.160 ± 0.009 | 0.367 |
|  | βNTP/TP | 0.100 ± 0.018 | 0.102 ± 0.008 | 0.863 |
|  | Pi/TP | 0.059 ± 0.007 | 0.055 ± 0.004 | 0.053 |
|  | pH | 7.034 ± 0.009 | 7.034 ± 0.005 | 0.946 |
| Right occipito-parietal white matter | PCr/TP | 0.164 ± 0.008 | 0.163 ± 0.013 | 0.874 |
|  | βNTP/TP | 0.101 ± 0.010 | 0.109 ± 0.009 | 0.087 |
|  | Pi/TP | 0.062 ± 0.006 | 0.055 ± 0.006 | 0.004 |
|  | pH | 7.041 ± 0.013 | 7.026 ± 0.008 | 0.012 |

Abbreviation: SD, standard deviation; PCr, phosphocreatine; TP, total phosphorus signal; ATP, nucleoside triphosphate; Pi, inorganic phosphate

^*^ Effect of site from mixed-effects linear regression

**Supplementary Table 2. Tissue-composition comparisons in each voxel among the groups**

| Site | Boston  (N = 22) | | Salt Lake City  (N =54) | | Oneway ANOVA |
| --- | --- | --- | --- | --- | --- |
| Diagnosis | HC  (N = 7) | BD  (N = 15) | HC  (N = 26) | BD  (N = 28) |  |
|  | Mean ± SD | Mean ± SD | Mean ± SD | Mean ± SD | p value |
| **Anterior Cingulate Cortex** | | | | |  |
| GM (%) | 66.7 ± 4.24 | 64.6 ± 7.82 | 76.2 ± 8.52 | 70.6 ± 6.91 | **0.0001** |
| WM (%) | 10.4 ± 4.67 | 11.1 ± 3.80 | 11.5 ± 4.52 | 10.6 ± 4.83 | 0.90 |
| CSF (%) | 23.0 ± 7.20 | 24.3 ± 9.04 | 12.4 ± 7.90 | 18.7 ± 7.07 | **<0.0001** |
| **Posterior Occipital Cortex** | | | | |  |
| GM (%) | 65.8 ± 4.67 | 65.0 ± 5.14 | 67.2 ± 5.38 | 65.9 ± 5.33 | 0.63 |
| WM (%) | 21.7 ± 9.10 | 22.8 ± 7.49 | 21.7 ± 7.68 | 19.6 ± 7.70 | 0.57 |
| CSF (%) | 12.4 ± 7.82 | 12.2 ± 6.67 | 11.1 ± 5.28 | 14.5 ± 7.34 | 0.29 |
| **Left Prefrontal WM** | | | | |  |
| GM (%) | 2.01 ± 1.20 | 4.90 ± 4.40 | 2.69 ± 2.53 | 2.47 ± 1.85 | **0.028** |
| WM (%) | 97.9 ± 1.24 | 94.4 ± 5.28 | 96.2 ± 4.72 | 96.9 ± 2.73 | 0.18 |
| CSF (%) | 0.14 ± 0.24 | 0.73 ± 1.48 | 1.10 ± 2.98 | 0.68 ± 1.38 | 0.71 |
| **Right Prefrontal WM** | | | | |  |
| GM (%) | 2.52 ± 3.01 | 3.59 ± 3.30 | 2.46 ± 2.53 | 2.72 ± 2.72 | 0.65 |
| WM (%) | 97.4 ± 3.18 | 95.7 ± 4.39 | 97.2 ± 3.16 | 97.0 ± 2.77 | 0.51 |
| CSF (%) | 0.07± 0.17 | 0.68 ± 1.34 | 0.33 ± 1.31 | 0.24 ± 0.43 | 0.47 |
| **Left Occipito-Parietal WM** | | | | |  |
| GM (%) | 2.63 ± 0.82 | 3.08 ± 3.04 | 2.74 ± 1.52 | 2.70 ± 1.26 | 0.91 |
| WM (%) | 95.6 ± 2.79 | 95.0 ± 4.99 | 95.8 ± 2.57 | 95.4 ± 2.49 | 0.88 |
| CSF (%) | 1.80 ± 2.48 | 1.87 ± 2.42 | 1.41 ± 1.72 | 1.94 ± 1.56 | 0.76 |
| **Right Occipito-Parietal WM** | | | | |  |
| GM (%) | 5.17 ± 4.54 | 3.93 ± 2.08 | 3.64 ± 3.33 | 3.32 ± 1.93 | 0.47 |
| WM (%) | 94.5 ± 4.80 | 95.3 ± 3.33 | 96.0 ± 4.07 | 96.2 ± 2.14 | 0.58 |
| CSF (%) | 0.36 ± 0.42 | 0.80 ± 1.72 | 0.37 ± 1.31 | 0.44 ± 0.55 | 0.68 |

Abbreviation: HC, healthy control; BD, bipolar disorder; SD, standard deviation; GM, gray matter; WM, white matter; CSF, cerebrospinal fluid

**Supplementary Table 3. Self-reporting total years of alcohol drinking in lifetime**

| Site | Boston  (N = 20^*^) | | Salt Lake City  (N =52^*^) | | T-test |
| --- | --- | --- | --- | --- | --- |
|  | Mean ± SD | | Mean ± SD | | p value |
| Total years of alcohol drinking | 24.0 ± 15.4 | | 16.2 ± 15.0 | | 0.052 |
| Diagnosis | HC  (N = 6) | BD  (N = 14) | HC  (N = 25) | BD  (N = 27) | Oneway  ANOVA |
|  | Mean ± SD | Mean ± SD | Mean ± SD | Mean ± SD | p value |
| Total years of alcohol drinking | 19.2 ± 18.3 | 26.1 ± 14.2 | 11.5 ± 13.5 | 20.5 ± 15.2 | 0.0252 |

^*^ Numbers are incomplete. Data of four subjects were not available.

Abbreviation: HC, healthy control; BD, bipolar disorder; SD, standard deviation

**Supplementary Table 4. Self-reporting days of alcohol drinking in a week during the last 6 months**

| Site | Boston  (N = 21^*^) | Salt Lake City  (N =54) | Wilcoxon  rank-sum  p value |
| --- | --- | --- | --- |
| Days of alcohol drinking in a week | | | 0.98 |
| 0 | 12 | 28 |  |
| 1 | 3 | 15 |  |
| 2 | 3 | 3 |  |
| 3 | 0 | 5 |  |
| 4 | 1 | 0 |  |
| 5 | 1 | 0 |  |
| 6 | 0 | 3 |  |
| 7 | 1 | 0 |  |

^*^ Numbers are incomplete. Data of one subject was not available.
